# Synchronous Oligometastatic Non-Small Cell Lung Cancer and Isolated Renal Cell Carcinoma: A Case Report and Literature Review

**DOI:** 10.7759/cureus.366

**Published:** 2015-10-27

**Authors:** Timothy K Nguyen, Alexander V Louie

**Affiliations:** 1 Department of Radiation Oncology, London Regional Cancer Program, Western University, London, Ontario, CA

**Keywords:** synchronous, non-small-cell lung cancer, renal cell carcinoma, metastatic, metastasis, oligometastasis, oligometastatic, concomitant, simultaneous, metastasectomy

## Abstract

A 58-year-old gentleman presenting with a progressive headache, visual disturbance, decreased appetite, and weight loss was found to have a localized clear cell carcinoma of the kidney and synchronous Stage IV non-small cell lung cancer with a solitary brain metastasis. This case illustrates the challenges in distinguishing between primary and metastatic disease in a patient with both renal cell carcinoma and lung cancer. We highlight the uncertainties in the diagnosis and management of this unique clinical scenario and the potential implications on prognosis.

## Introduction

The oligometastatic state is a clinical paradigm whereby cancer patients with limited metastases (i.e., 1 to 5) that are amenable to aggressive management through resection or ablative therapy may survive beyond expectations [1-2]. At the time of diagnosis, non-small cell lung cancer (NSCLC) and renal cell carcinoma (RCC) present with metastatic disease in approximately 56% and 30% of cases, respectively [3]. Renal metastases have been reported in 16-23% of metastatic NSCLC cases while, for metastatic RCC, the lungs are the most common site of metastasis representing up to 45% of cases [4-5]. In situations with radiographic evidence of both pulmonary and renal tumors, the diagnostic challenge becomes differentiating between primary neoplasms and metastatic disease. We report a case of oligometastatic NSCLC with a solitary brain metastasis in the setting of a synchronous primary renal neoplasm. Informed patient consent was obtained prior to the drafting and submission of this report.

## Case presentation

### Initial assessment

We present a case of a 58-year-old, right-handed gentleman with a medical history of Type II diabetes, obstructive sleep apnea, gastroesophageal reflux disease, asthma, gout, anxiety, and depression. He was an ex-smoker with a 40 pack-year smoking history. His oncologic family history was significant for his father dying from bladder cancer.

In the spring of 2014, the patient initially presented to his local emergency room with progressive frontotemporal headaches, a right visual field deficit, decreased appetite, weight loss, and a fall without sustaining traumatic injuries. On a review of systems, no respiratory, genitourinary, or additional neurological symptoms were described aside from increased daytime urinary frequency.

Physical examination revealed an obese gentleman (BMI of 41) with normal vital signs. The patient was alert, oriented to person, place and time, and ambulatory with a normal gait. Cranial nerve exam revealed right visual field deficits. Auscultation of the lungs revealed clear, equal air entry bilaterally without any adventitious sounds appreciated. No masses, organomegaly, or pelvic lymphadenopathy were appreciated on the abdominal exam, but this was limited due to his large body habitus. Digital rectal exam revealed a small, smooth, firm prostate without rectal masses.

**Figure 1:**
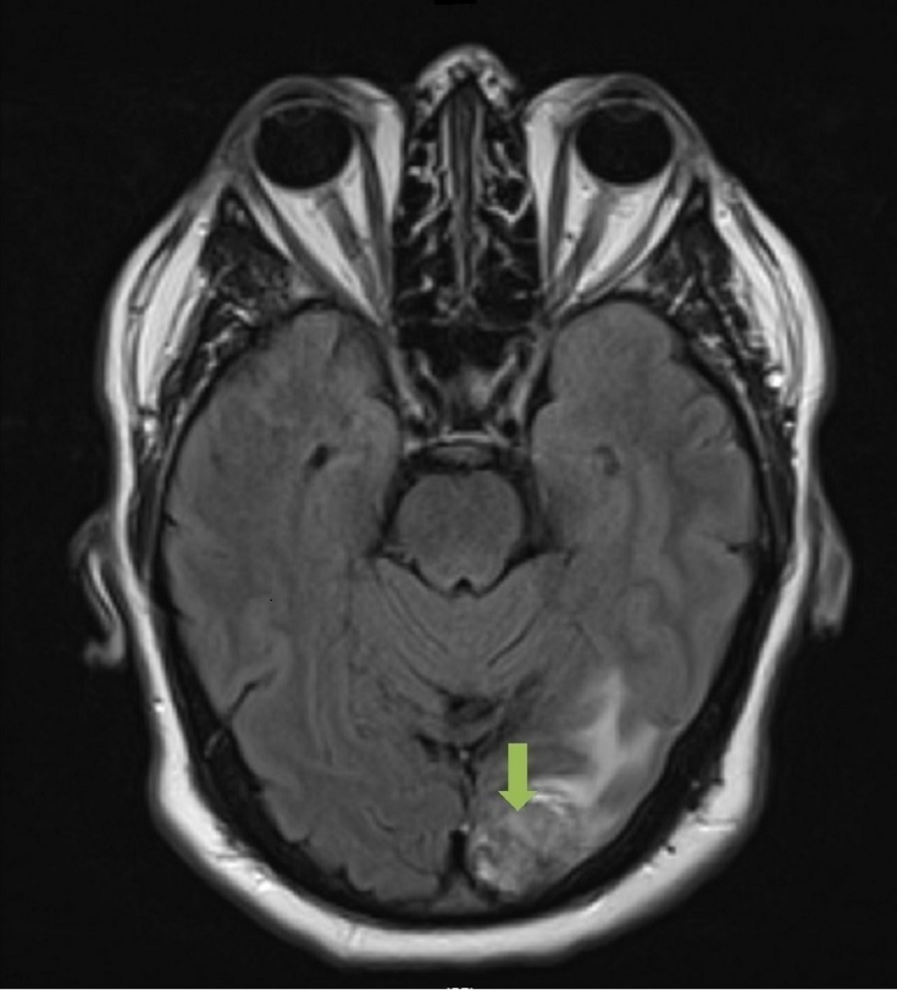
Preoperative MRI of the Head Axial slice. T2-weighted image that demonstrates a solitary 2.9 cm left occipital lobe adenocarincoma, metastatic from a lung primary.

A CT and MRI of the head was completed with the latter revealing a heterogeneous mass in the left occipital lobe with solid and cystic components measuring 2.9 cm. There was associated cerebral edema with a 0.2 cm right-sided midline shift and no hydrocephalus identified (Figure 1).

### Initial management

The leading differential diagnosis at the time was a primary brain malignancy, and accordingly, referral to neurosurgery at the same tertiary hospital was arranged. The patient subsequently underwent left occipital craniotomy and resection of the mass within five weeks of the initial referral. Pathology returned as metastatic adenocarcinoma, suggestive of a lung primary as it stained positive for cytokeratin 7 and TTF-1 and negative for cytokeratin 20. Adjuvant whole-brain radiotherapy was delivered to a dose of 30 Gy in 10 fractions, which was completed within six weeks from the time of surgery. The start date of radiotherapy was delayed by a couple of weeks as a result of a postoperative wound infection that was managed with oral antibiotics. During this time, the patient had a staging CT scan in the community encompassing the thorax, abdomen, and pelvis, which revealed a 5 cm left upper pole renal mass without other sites of disease. This report was not available in the patient’s record; however, the dictated notes from the medical team indicated that metastatic renal cell carcinoma was the provisional diagnosis.

**Figure 2:**
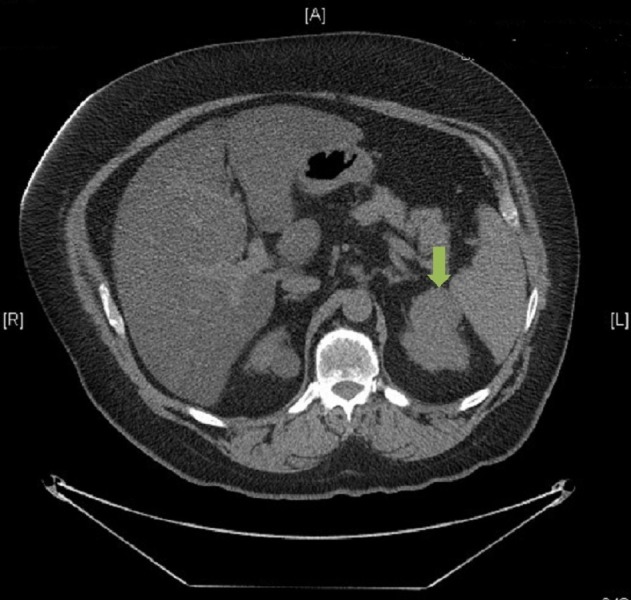
Preoperative CT of the Abdomen Axial slice. Contrast-enhanced image that demonstrates a 4.9 cm isolated clear cell renal cell carcinoma involving the upper pole of the left kidney.

**Figure 3:**
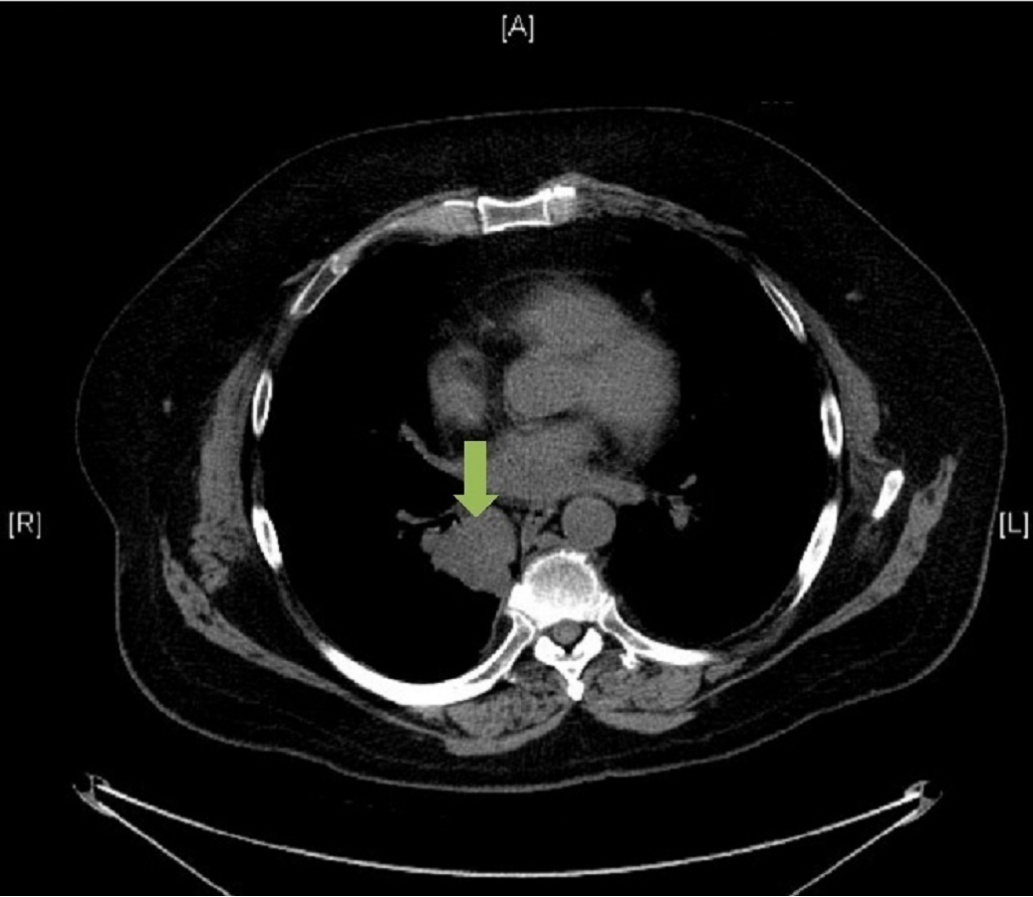
Preoperative CT of the Thorax Axial slice. Contrast-enhanced image that demonstrates a 2.1 cm primary papillary adenocarcinoma of the right lower lobe of the right lung.

Concomitant with the planning and delivery of whole brain radiotherapy, the patient was also seen and assessed by the urology team at the same institution. Restaging CT imaging of the thorax, abdomen, and pelvis was completed two weeks after completion of radiotherapy, which revealed an exophytic solid renal mass arising from the upper pole of the left kidney measuring 4.9 cm. In addition, there were now two other areas of concern: a 3.1 cm heterogeneous pancreatic mass containing cystic components and a 2.1 cm pulmonary nodule in the lower lobe of the right lung. Both lesions, radiographically, were thought to be metastases from a primary renal cell carcinoma. Considerations for management included cytoreductive surgery, up-front Sunitinib, or enrolling the patient on a clinical trial (Figures 2-3).

However, before the patient could return to further discuss management options, an MRI scan of the head, completed two months after finishing radiotherapy to assess treatment effect, showed evidence of an intracranial abscess in the previous operative bed. Specifically, there was an interval development of a 4.2 cm rim-enhancing fluid signal in the post-surgical bed. Urgently, the patient was brought to the operating room for a left occipital craniectomy for evacuation and resection of the abscess. The patient recovered well without any additional complications, although did not wish to seek any further medical care at his local institution.

### A second opinion

The patient’s primary care team in the community referred the patient to the multidisciplinary genitourinary oncology team at our center for a second opinion regarding management of the patient’s remaining disease. Following a review of the original imaging and pathology reports, the possibility of metastatic lung cancer (given the immunohistochemistry of the resected brain lesion favoring a lung primary) or synchronous primary malignancies was proposed. A PET-CT scan was ordered, which re-demonstrated the pulmonary mass in the lower lobe of the right lung, now measuring 3.0 cm with an SUV uptake of 9. A 1 cm non-18-FDG-avid subcarinal lymph node was also identified. The renal lesion measured 4.2 cm and was not 18-FDG-avid. A nodule in the pancreatic tail was thought to be a benign process, given its cystic appearance, stable size, and lack of 18-FDG uptake.

The case was also presented at our thoracic multidisciplinary team conference with two recommendations provided. First, it was advised to complete the diagnostic workup, including biopsies of both the lung and renal masses, in addition to sampling the mediastinal lymph nodes. Second, depending on the results of the investigations, treatment should be considered in accordance with the oligometastatic paradigm, which could involve sequential resection of the pulmonary and renal tumors.

An ultrasound-guided biopsy of the kidney mass confirmed renal cell carcinoma (clear cell). The sample stained positive for CKAE1/AE3 and vimentin, while staining negative for CK7, CK20, and TTF-1. Through an endobronchial ultrasound (EBUS)-guided biopsy, tissue was obtained from the right lower lobe mass and a paraesophageal (level 8) lymph node. The lymph node was negative for malignancy while the biopsy from the mass showed the presence of neoplastic cells. Given the limited sample, pathologists were unable to distinguish definitively between a well-differentiated adenocarcinoma and a neuroendocrine tumor at that time. It was evident, however, that the lung and kidney pathology were distinctly different.

### Management

In accordance with recommendations from both our multidisciplinary teams, the patient proceeded with radical intent treatment, first undergoing a video-assisted transthoracic surgery (VATS) right lower lobe lobectomy within two months of initial assessment at our center. Pathology revealed a unifocal tumor measuring 4.5 cm and was characterized as a Grade 2 papillary adenocarcinoma with negative margins. Eight lymph nodes were removed, and one peribronchial lymph node was found to be positive for malignancy with extranodal extension. Final lung pathologic staging was T2aN1.

Postoperatively, the patient met and discussed with the medical oncology team regarding the benefits of adjuvant chemotherapy in the setting of N1 and oligometastatic disease. Given the unclear benefit systemic therapy might provide for completely resected oligometastatic disease in a patient with moderate comorbidities, adjuvant chemotherapy was ultimately declined by the patient despite it being offered.

 

 

Restaging CT of the abdomen and pelvis was completed three months following VATS resection and demonstrated that the kidney mass had grown in size to 6.4 cm since previous imaging. The patient subsequently underwent a left partial nephrectomy three months later. Pathology revealed a unifocal 6.0 cm tumor in the upper pole of the left kidney, which was resected with negative margins. Histologically, the tumor was consistent with the previous biopsy, characterized as a clear cell renal cell carcinoma. Regional lymph nodes were not sampled. The final pathologic staging was pT1bNx, and no additional adjuvant treatment was felt to be indicated.

The patient was seen in follow-up three months following resection of the renal mass, which was 10 and 16 months from previous pulmonary and intracranial resections, respectively. He recovered well without surgical complications and was found to have no clinical evidence to suggest disease recurrence or progression. Repeat CT imaging of the head, thorax, abdomen, and pelvis revealed no evidence of recurrent or residual disease. Moving forward, the patient will continue with routine CT surveillance every three to six months. 

## Discussion

Synchronous primary malignancies are complex cases that present with diagnostic, prognostic, and therapeutic uncertainties, particularly if there has already been metastatic spread from one or both of the primary sites. We report on a case of oligometastatic adenocarcinoma of the lung with a solitary brain metastasis that presents synchronously with a primary clear cell renal cell carcinoma. The challenges surrounding this case stem from the propensity of lung cancer to metastasize to the kidneys and brain, and likewise, the ability for kidney cancers to metastasize to the lungs and brain. In such situations, histologic confirmation with immunohistochemistry can help distinguish between metastatic and primary disease. Given the paucity of evidence-based standards to guide treatment decisions in these clinical scenarios, we propose that such cases be discussed in the context of the relevant multidisciplinary tumor boards.

There have been a limited number of published cases reporting on the diagnostic and therapeutic approach to synchronous primary pulmonary and renal neoplasms. Similar to our case, a report from the Netherlands described a patient with a TTF-1 positive adenocarcinoma of the right lower lobe of the lung invading into the thoracic vertebrae that presented synchronously with an 18-FDG-negative lesion in the right kidney [6]. Initially, the case was considered to be Stage IV NSCLC with the renal mass representing a distant metastasis and, as such, was managed with an expectative approach. In the context of a second opinion and multidisciplinary reevaluation, however, a provisional diagnosis of two synchronous primary neoplasms was made. The lung cancer was managed with two cycles of neoadjuvant chemotherapy (cisplatin and pemetrexed) followed by surgical resection. The kidney cancer was managed with surveillance, given its stability in size, until it was later resected at the patient’s request. The patient was disease-free at the six years following radical treatment, again highlighting the difficulty and importance in differentiating between primary versus metastatic disease.

Distinguishing between a renal metastasis of lung origin and a primary RCC (or vice versa) can be a difficult task. The use of PET imaging can be useful in identifying metastatic deposits in the kidneys, which tend to be 18-FDG-avid. However, a lack of hypermetabolic activity does not rule out the possibility of a primary RCC. PET imaging has been described to have a low sensitivity to detect primary renal malignancies, which was consistent with the experience of Mazouz, et al. and with our own findings in the present case [6-7]. Although renal metastases are typically bilateral and multi-focal, they can also present as a large, solitary metastasis in breast, lung, and colorectal patients [8]. Thus, obtaining pathology is advised in patients amenable for biopsy. In addition, only 1-3% of patients with metastatic RCC present with a solitary metastasis [9]. When considered alongside additional supportive clinical evidence, this point may steer clinicians away from a diagnosis of metastatic RCC in cases of synchronous primary neoplasms with a solitary metastasis.

In a retrospective review of percutaneous core biopsies for renal masses, unnecessary surgery was avoided in 30% of cases in which biopsies revealed benign tumors or non-surgical metastatic disease [10]. Sanchez-Ortiz, et al. conducted a retrospective review of 100 patients with a renal mass in the setting of a non-renal primary malignancy. Fifty-nine percent of renal masses were malignant primary tumors, 19% were metastases (lung and lymphoma being the most common primary sites), 12% were benign primary tumors, and 10% had non-diagnostic biopsies. All cases of renal metastasis occurred in the setting of clinical progression or radiographic evidence of other metastases from the non-renal primary cancer [11]. The authors contend that for the majority of patients with a renal mass in the context of a clinically localized non-renal malignancy, the renal tumor is unlikely to be metastatic disease. Once pathology is obtained, immunohistochemical techniques can help direct diagnosis, with TTF-1 staining positive in approximately 76% of lung adenocarcinomas [12].

In the oligometastatic setting, there have been several publications that demonstrate a prolonged survival when pursuing metastasectomies in intracranial, hepatic, and pulmonary metastases [13-15]. However, randomized data to support a benefit in overall survival with radical treatment for oligometastatic disease has only been demonstrated in the solitary brain metastasis setting [16]. As the body of literature on the management of oligometastatic disease continues to grow, heightened attention towards its implications on prognosis and treatment will be vital in ensuring high-quality patient care.

## Conclusions

In conclusion, we present a novel case of a 58-year-old gentleman presenting with a synchronous localized renal cell carcinoma in the setting of oligometastatic NSCLC with a solitary brain metastasis. In accordance with the oligometastatic paradigm, the patient is alive, disease-free, and well 14 months from the initiation of his treatment. Management of synchronous primary neoplasms, particularly in the oligometastatic setting, relies on a thorough diagnostic workup and careful consideration of all possible clinical scenarios, which should occur in the context of a multidisciplinary team approach.  
